# ECHS1, an interacting protein of LASP1, induces sphingolipid-metabolism imbalance to promote colorectal cancer progression by regulating ceramide glycosylation

**DOI:** 10.1038/s41419-021-04213-6

**Published:** 2021-10-06

**Authors:** Rui Li, Yanyu Hao, Qiuhan Wang, Yuan Meng, Kunhe Wu, Chaoqun Liu, Lijun Xu, Ziguang Liu, Liang Zhao

**Affiliations:** 1 Department of Pathology, Shunde Hospital, Southern Medical University (The First People’s Hospital of Shunde), Foshan, China; 2 Department of Pathology & Guangdong Province Key Laboratory of Molecular Tumor Pathology, School of Basic Medical Sciences, Southern Medical University, Guangzhou, China; 3 Department of Pathology, The Second People’s Hospital of Longgang District, Shenzhen, China; 4 Department of Pathology, Guangdong Women and Children Hospital, Guangzhou, Guangdong 511442 China

**Keywords:** Cancer metabolism, Colorectal cancer

## Abstract

Sphingolipid metabolic dysregulation has increasingly been considered to be a drug-resistance mechanism for a variety of tumors. In this study, through an LC–MS assay, LIM and SH3 protein 1 (LASP1) was identified as a sphingolipid-metabolism-involved protein, and short-chain enoyl-CoA hydratase (ECHS1) was identified as a new LASP1-interacting protein through a protein assay in colorectal cancer (CRC). Gain- and loss-of-function analyses demonstrated the stimulatory role played by ECHS1 in CRC cell proliferation, migration, and invasion in vitro and in *vivo*. Mechanistic studies of the underlying tumor-supportive oncometabolism indicate that ECHS1 enables altering ceramide (Cer) metabolism that increases glycosphingolipid synthesis (HexCer) by promoting UDP-glucose ceramide glycosyltransferase (UGCG). Further analysis showed that ECHS1 promotes CRC progression and drug resistance by releasing reactive oxygen species (ROS) and interfering mitochondrial membrane potential via the PI3K/Akt/mTOR-dependent signaling pathway. Meanwhile, the phenomenon of promoting the survival and drug resistance of CRC cells caused by ECHS1 could be reversed by Eliglustat, a specific inhibitor of UCCG, in vitro and in vivo. IHC assay showed that ECHS1 was overexpressed in CRC tissues, which was related to the differentiation and poor prognosis of CRC patients. This study provides new insight into the mechanism by which phospholipids promote drug resistance in CRC and identifies potential targets for future therapies.

## Background

Colorectal cancer (CRC) is one of the most common malignant tumors threatening human health worldwide [1]. Chemotherapy is the most commonly used adjuvant and conversion therapy for patients with advanced CRC. Despite significant progress in chemotherapy, inherent or acquired chemoresistance, especially multidrug resistance (MDR), is the main obstacle to this development, leading to inefficient cancer-cell killing and subsequent patient relapse [2]. Therefore, it is essential to understand the biological mechanisms underlying MDR more comprehensively to benefit patients with CRC or other tumors.

LIM and SH3 protein 1 (LASP1) was identified as a tumor-promotion-related gene and its abnormal expression has been reported in several tumor types, including breast cancer [3], ovarian cancer [4], hepatocellular carcinoma [5], and CRC [6]. On the basis of our previous study, we found that LASP1 was highly overexpressed in CRC tissues which is positively associated with lymph node and distant metastasis [7]. By using comparative proteomics methods to identify LASP1-related proteins involved in regulating tumor progression, a series of proteins related to cytoskeleton [8], epithelial–mesenchymal transition (EMT), and metabolism is found to interact with LASP1 to promote aggressive phenotype of CRC cells. Moreover, microRNAs, including miR-1 [9], miR-133a [10], and miR-145 [11], suppress colon-cancer cell invasion and metastasis by targeting LASP1. Although there have been many researches on the molecular mechanism of LASP1 promoting the progression of CRC, it promotes cell metabolism, especially regarding sphingolipid metabolism, which has not been elucidated in detail. However, the molecular mechanism underlying this interaction, especially regarding sphingolipid metabolism, has not been elucidated in detail.

Short-chain enoyl-CoA hydratase (ECHS1) is responsible for the second step of hydration of fatty-acid β oxidation(FAO) and its activity was first observed by Del Campillo and Stern in cow hearts and livers, and the human ECHS1 cDNA clones were first isolated in 1993 [12]. The ECHS1 gene mapped to human chromosome 10q26.2–q26.3, which encodes eight exons, including 50 and 30 untranslated regions containing exons I and VIII, respectively [13]. The expression of ECHS1 has been identified in numerous types of cancer cells or patient tissues by gene or proteomic expression profiling. It was reported that the expression of ECHS1 was significantly increased in colorectal [14, 15], liver [16], and gastric [17] cancers. Although evidence suggests a potential role of ECHS1 involved in tumor occurrence and development, the underlying mechanisms have not been elucidated to date. Most importantly, in addition to abnormal FA-oxidation (FAO) synthesis, the relevance of phospholipid metabolism associated with ECHS1 and cancer-cell function is unknown.

In this study, we found that LASP1 colocalizes with ECHS1 in CRC cells and is associated with sphingomyelin metabolism to induce apoptosis and drug resistance. The expression and function of ECHS1 in CRC was studied, and the associated signaling pathway was investigated. The current study helps to elucidate the relationship between multidrug resistance and phospholipid metabolism and provides a new insight for overcoming clinical CRC chemotherapy resistance.

## Materials and methods

### Cell-line preparation

The CRC cell lines RKO, HCT116, HCT15, SW620, SW480, LS174T, and CACO2 were obtained from CBCC (Shanghai, China) and maintained as previously described [6]. All cells were cultured in RPMI-1640 medium (KeyGen, Keygentec, JiangSu, China) containing 10% fetal bovine serum (Gibco-BRL, Invitrogen, Paisley, UK) in a humidity of 5% CO2 at 37 °C.

### Tumor-tissue samples

The six pairs of primary colorectal cancer tissues and their paired adjacent noncancerous tissues were obtained from the Tumor Tissue Bank of Nanfang Hospital. All cases were from patients diagnosed with primary CRC and undergoing surgery at Nanfang Hospital in 2017–2019. The study was approved by the Ethics Committee of Southern Medical University, and all aspects of the research had obtained informed consent and in accordance with the Declaration of Helsinki.

### Animals and tumor-growth assay

The Institutional Animal Care and Use Committee of Southern Medical University (Guangzhou, China) approved the animal experiments involved. All animal procedures were in accordance with the Helsinki Declaration. BALB/c nude mice (female, 3–5 weeks, 13–15 g) were obtained from Southern Medical University Experimental Animal Center and kept in an SPF animal room with free access to clean food and water and possible adverse events were monitored. During housing, animals were monitored twice daily for health status. For subcutaneous tumor tests for drug sensitivity, the mice were divided into four groups (*n* = 20): (1) injected with LV-ctrl-HCT116 cells (*n* = 5); (2) injected with LV-ctrl-HCT116 cells and injected intraperitoneally with Oxaliplatin (1.5 mg/kg, MedChem Express, HY-17371) every two days (*n* = 5); (3) injected with LV-ECHS1-HCT116 cells and injected intraperitoneally with Oxaliplatin (1.5 mg/kg, MedChem Express, HY-17371) every two days (*n* = 5); and (4) injected with LV-ECHS1-HCT116 cells and injected intraperitoneally with Oxaliplatin (1.5 mg/kg, MedChem Express, HY-17371) every two days and received daily intraperitoneal injections of Eliglustat (60 mg /kg, MedChem Express, HY-14885) [18] (*n* = 5). Three mutual experimenters are responsible for grouping using a blinding and randomization method, processing, and data collection.

For the tumor-growth assay, 5 × 10^6^ cells were injected into the subcutaneous tissue of the back of the mice. After four weeks, the nude mice were anesthetized with diethyl ether and sacrificed by cervical dislocation before tumors reached 1500 mm^3^ in volume. The xenograft tumors were harvested for subsequent histological study. The formula (volume (mm^3^) = width^2^ (mm^2^) × length (mm)/2) was used to calculate the tumor volume.

### Lipid extraction

For sample preparation, 1500 μL of chloroform methanol mixed solution (2:1) (precooled at −20 °C) and 100-mg glass beads were added into the sample. The samples were frozen in liquid nitrogen and then lysed ultrasonically at a frequency of 50 Hz. After adding ddH2O and standing on ice for 10 min, centrifuge at 12,000 rpm for 5 min at room temperature. Take the lower layer and add 1000 μL of chloroform methanol mixed solution (2:1) (precooled at −20 °C), then centrifuge at 12,000 rpm at room temperature for 5 min. Remove the lower layer of liquid and add 200 μL of isopropanol to dissolve it and filter it with a membrane to prepare a sample for LC–MS [19].

### LC–MS conditions

The chromatographic separation was performed in a Thermo Ultimate 3000 system that was equipped with ACQUITY UPLC^®^ BEH C18(100 × 2.1 mm, 1.7 µm, Waters) column and kept at 50 °C. The ESI–MSn experiments were performed on the Thermo Q Exactive Focus mass spectrometer, and the spray voltages of positive and negative modes were 3.5 kV and −2.5 kV, respectively.

### Western blot analysis

Western blot analysis was performed to evaluate protein expression(30–60 mg) in the presence of antibodies to ECHS1 (Proteintech, 66117-1-Ig, 1:1000), LASP1 (Proteintech, 10515-1-AP, 1:1000), Phospho-Akt (CST, 4060, 1:1000), PI3K (CST, 4249, 1:1000), Phospho-mTOR (CST,5536, 1:500), Beclin1 (CST, 3495, 1:1000), ATG5 (CST, 12994 S, 1:500), LC3I/II (Novus, NB600-1384, 1:500), Caspase 3 (Proteintech, 66470-2-Ig, 1:500), UGCG (SAB, 35753, 1:300), BCL2 (Proteintech, 12789-1-AP, 1:500), HA-Tag (CST, 3724 S, 1:1000), Flag-Tag (CST,8146 S, 1:1000), Tubulin (Zsbio, TA-10, 1:1000), and GAPDH (Zsbio,TA-08, 1:1000).

### Immunohistochemistry (IHC)

The CRC specimens were incubated overnight using primary antibodies against LASP1 (Proteintech, 10515-1-AP, 1:500) and ECHS1 (Proteintech, 66117-1-Ig, 1:500) at 4 °C. Mayer’s hematoxylin was used for nuclear counterstaining. In this study, each slide was reviewed by three blinded pathologists.

### Statistical analysis

SPSS statistical software version 19.0 (SPSS; Chicago, USA) was used to analyze the data. The *t*-test and one-way ANOVA were applied for RT-PCR. Spearman rank-correlation test was used to determine the correlation between ECHS1 and LASP1. Pearson chi-square (*χ*^2^) was applied to determine the correlation between the expression of ECHS1 and histopathological factors. The independent *t*-test was used for subcutaneous tumor detection. Kaplan–Meier chart was used to estimate the prognostic correlation of ECHS1 in univariate analysis. The statistical significance was established at *P* < 0.05. All the experiments were carried out in triplicate.

## Results

### LASP1 is associated with sphingomyelin metabolism and regulates ECHS1 in CRC cells

**Fig. 1:**
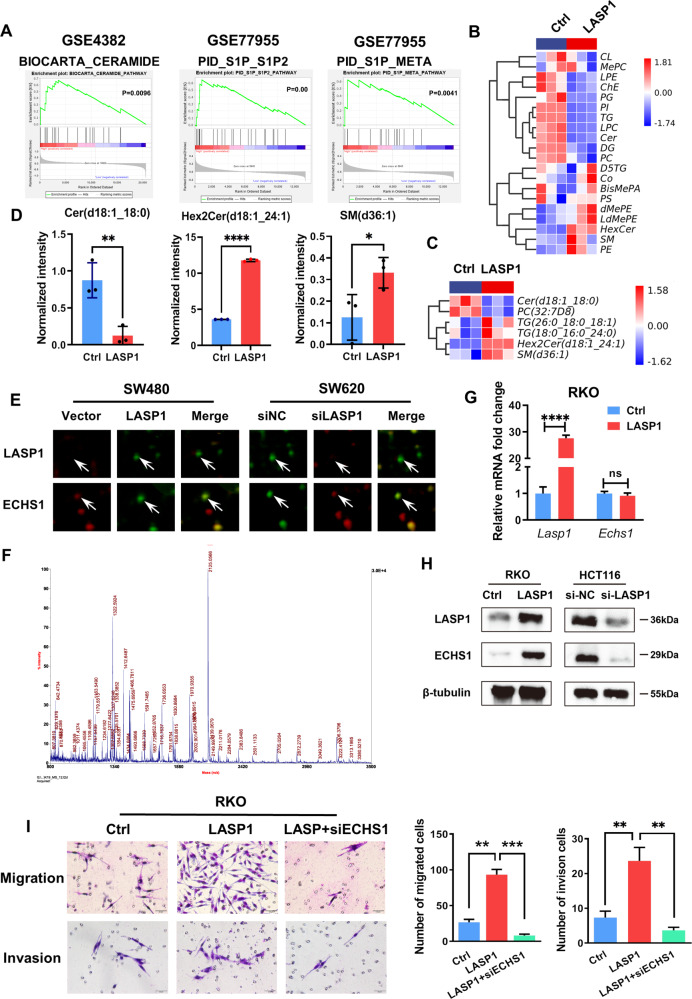
LASP1 is associated with sphingomyelin metabolism and regulates ECHS1 in CRC cells. **A** GSEA of the top 100 upregulated LASP1-related pathways, including biocarta ceramide, PID_S1P_S1P2 and PID_S1P_META. **B** Heatmap of the LC–MS assay for RKO-LASP1 and control cells. **C** Heat map depicting the differentially expressed lipomics for RKO-LASP1 and control cells. **D** Histogram of c. The asterisk (*) indicates *P* < 0.05. The double asterisk (**) indicates *P* < 0.01. The quadra asterisk (****) indicates *P* < 0.0001. **E** Fluorescence images show the two differentially expressed protein spots in DIGE analysis. The interacting protein spots are indicated (white arrows). **F** MS of in-gel trypsin digests of the protein and analysis of the depicted peptide spectrum resulted in the identification of ECHS1. **G**, **H** RT-PCR (**G**) and Western blotting (**H**) were performed to detect the expression of ECHS1 and LASP1 in LASP1-overexpressing and LASP1-knockdown cells. **I** Representative figures and data of the Transwell assay for LV-control-, LV-LASP1-, and LV-LASP1 plus siRNA-ECHS1-transfected RKO cells. Bars in the right panel represent the number of migrated and invaded cells.

We first evaluated the role of LASP1 in CRC lipid metabolism. As shown in Fig. 1A, gene-set enrichment analysis (GSEA) demonstrated a strong enrichment of phospholipids, including ceramide (GSE4382), sphingosine kinase/sphingosine 1-phosphate (S1P_S1P2) (GSE77955), and S1P_META (GSE77955). Next, liquid chromatography–mass spectrometry technology (LC–MS) was applied to detect the lipidomics of LASP1-overexpressing CRC cells (RKO-LASP1) and control cells. Notably, as shown in Fig. 1B, abnormal metabolism of phospholipids, including increased ceramide and decreased sphingomyelin, was found in RKO-LASP1 cells compared with the control group. Specifically, the expression of ceramide (d18:1_18:0) decreased, whereas the expression of Hex2Cer (d18:1_24:1) and SM (d36:1) increased (Fig. 1C, D). Hexose-linked glycoceramides (HexCer), including galactosylceramide (GalCer) and glucosylceramide (GluCer).

To further study the underlying mechanism of LASP1-mediated CRC-cell malignance, 2-D DIGE assay was used to screen the differentially expressed proteins in SW480/ SW620 cells transfected with LASP1 siRNA or control siRNA (Fig. S1). The results demonstrated that ECHS1, an enzyme involved in lipid metabolism, was one of the candidates for LASP1-modulated proteins (Fig. 1E, F). RT-PCR (Fig. 1G) and Western blotting (Fig. 1H) results showed that LASP1 regulates ECHS1 at the protein, rather than the RNA level. Furthermore, as shown in Fig. 1I, the results of the Transwell assay demonstrated that the ability of LASP1 to promote CRC-cell migration and invasion was related to its regulation of ECHS1.

### ECHS1 is essential for LASP1-mediated sphingomyelin-metabolism imbalance by interacting with SH3 domain of LASP1 in CRC

**Fig. 2:**
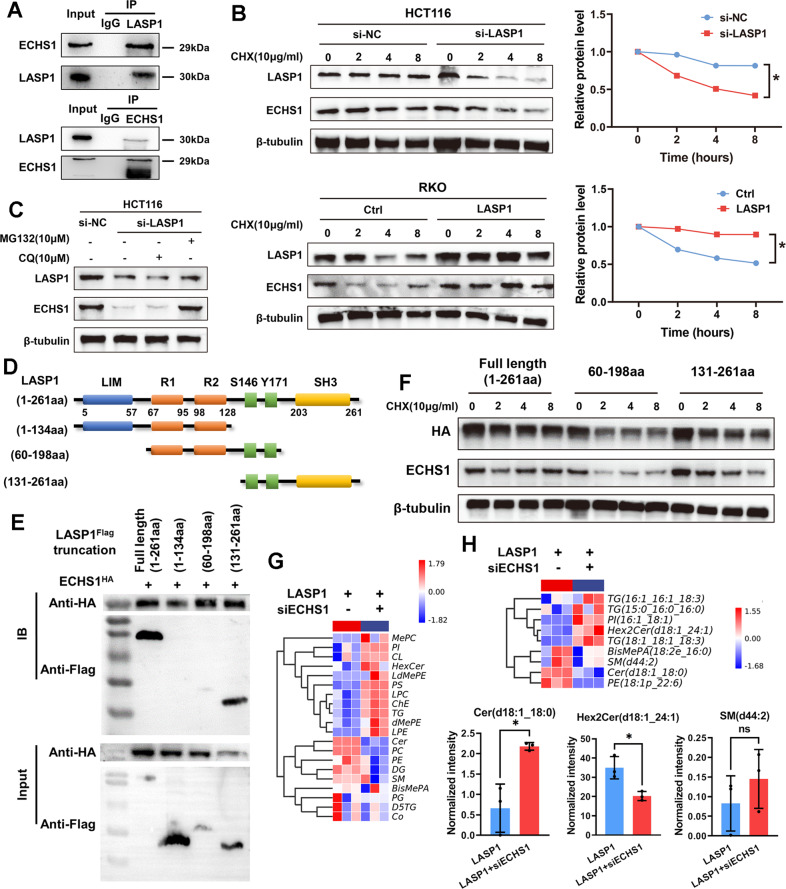
ECHS1 is essential for LASP1-mediated sphingomyelin-metabolism imbalance by interacting with SH3 domain of LASP1 in CRC. **A** Immunoprecipitation-assay analysis of endogenous interaction between ECHS1 and LASP1 in SW480 cells. **B** Western blot assay of LASP1 and ECHS1 protein and quantification of ECHS1 relative level at the indicated time in HCT116 cells transfected with siLASP1 or siNC (upper figure) or RKO cells transfected with LV-LASP1 or LV-Ctrl (lower figure) after CHX treatment to block protein synthesis. **C** Western blot assay of the level of LASP1 and ECHS1 protein with application of chloroquine (10uM) or MG132 (10uM) after 8 h. **D** Schematic presentation of LASP1 structure features. The structures of truncated LASP1 are listed behind those of the full-length proteins. **E** Western blotting results indicate the binding domain of ECHS1 and LASP1 in 293 T cells. **F** Western blot assay of HA and ECHS1 protein in 293 T cells transfected with plasmid HA-LASP1 full length (1–261aa), HA-LASP1 (60–199aa), or HA-LASP1(131–261aa) after CHX treatment to block protein synthesis. **G** Heatmap of the LC–MS assay for RKO-LASP1 and RKO-LASP1 transfected with siECHS1 cells. **H** Heatmap and histogram of depicting the differentially expressed lipomics for RKO-LASP1 and RKO-LASP1 plus siRNA-ECHS1 cells.

The interaction between LASP1 and ECHS1 was further verified by immunoprecipitation (Co-IP) assays (Fig. 2A) using protein extraction of SW480 cells. As shown in Fig. 2B, when protein synthesis is blocked by CHX, ECHS1 gradually degrades with time. CRC cells were transfected with plasmid-encoding LASP1 or control plasmid, and the degradation of ECHS1 protein was monitored after CHX treatment. Compared with the control group, LASP1 evidently prevented ECHS1 degradation, thus prolonging its half-life. Furthermore, western blot assay showed that chloroquine can reverse the degradation of ECHS1 protein prevented by LASP1 instead of MG132, indicating that LASP1 inhibits the degradation of ECHS1 by inhibiting its hydrolysis in the proteasome (Fig. 2C). Given that LASP1 contains the N-terminal LIM domain and the C-terminal SH3 domain (Fig. 2D), therefore, we constructed full-length Flag-LASP1 (1–261a), Flag-LASP1 (1–131 aa), Flag-LASP1 (60–198 aa), Flag-LASP1 (131–261 aa), and HA-ECHS1-overexpression vectors and transfected 293 T cells. As shown in Fig. 2E, ECHS1 was detected by Co-IP assay only after Flag-LASP1 (132–261aa) and full-length Flag-LASP1 (1–261aa) were transfected into 293 T cells, which indicated that ECHS1 might directly bind to the SH3 domain of LASP1. Meanwhile, as shown in Fig. 2F, western blot assays showed that LASP1 prevented ECHS1 degradation induced by CHX only after transfection with plasmids containing LASP1–SH3 domain.

To verify whether LASP1 regulates phospholipid metabolism in CRC through ECHS1, we knocked down ECHS1 in LASP1-overexpressing cells and detected its lipidomics by LC–MS. As shown in Fig. 2-G, after knocking down ECHS1, the imbalance of phospholipid metabolism caused by LASP1 overexpression was restored. Meanwhile, as shown in Fig. 2-H, single-fatty-acid analysis showed that ceramide (d18:1_18:0) was increased and Hex2Cer (d18:1_24:1) and SM (d44:2) were decreased when ECHS1 was knocked down in LASP1-overexpressing cells. Generally, ECHS1 interacted with LASP1 and regulated ceramide to glycosylated ceramide in CRC.

### ECHS1 promotes the conversion of ceramide to glycosylated ceramide by regulating UGCG

**Fig. 3:**
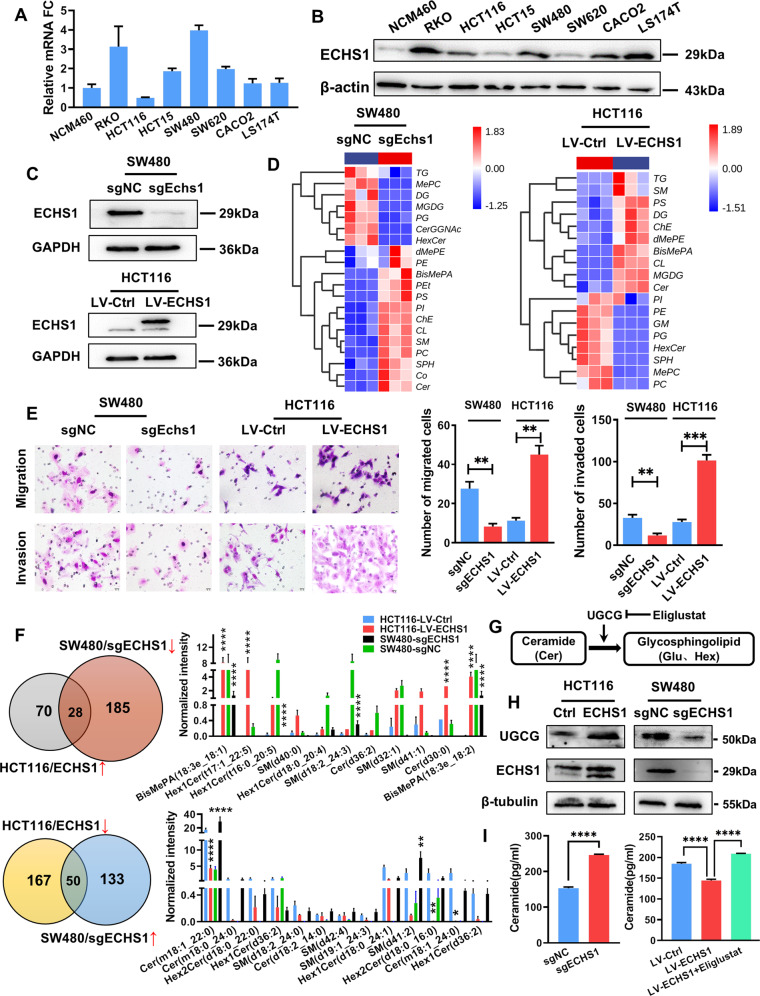
ECHS1 promotes the conversion of ceramide to glycosylated ceramide by regulating UGCG. **A**, **B** RT-PCR (**A**) and Western blot analysis (**B**) were used to detect the expression of ECHS1 in CRC cell lines. **C** Western blot analysis showed that ECHS1-knockout and -overexpression cells were constructed. **D** Left panel: heatmap of the LC–MS assay for SW480sgECHS1 and SW480sgNC cells. Right panel: heatmap of the LC–MS assay for HCT116-LV-ECHS1 and HCT116-LV-Ctrl cells. **E** Representative figures and data of the Transwell assay for SW480sgECHS1, SW480sgNC, and HCT116-LV-ECHS1 and HCT116-LV-Ctrl cells. Bars in the right panel represent the number of migrated and invaded cells. The triple asterisk (***) indicates *P* < 0.001. **F** Upper panel: pi chart of lipids associated with the elevation of endogenous ECHS1. Bars in the right panel represent the lipids associated with phospholipid metabolism. Lower panel: pi chart of lipids associated with the reduction in endogenous ECHS1. Bars in the right panel represent the lipids associated with phospholipid metabolism. **G** Schematic diagram of UGCG regulating ceramide glycosylation. **H** Western blot analysis showed the expression of UGCG when endogenous ECHS1 changed in CRC cells. **I** Histogram of ELISA results showing the expression of endogenous ceramide when ECHS1 was overexpressed or knocked out in CRC cells.

ECHS1 is generally considered to be involved in the hydration step of fatty-acid oxidative phosphorylation, but its expression and role in CRC are still controversial. As shown in Fig. 3A and B, ECHS1 was highly overexpressed in CRC cells either in mRNA or protein level compared with nonneoplastic cell line NCM460. To further investigate the biological behaviors of ECHS1, CRISPR–Cas9 was used to construct ECHS1-knockout cells (SW480-sgECHS1), and lentivirus was used to build ECHS1-overexpressing cells (HCT116-LV-ECHS1) (Fig. 3C). Meanwhile, as shown in Fig. 3E, Transwell assays showed that overexpression of ECHS1 dramatically enhanced the migration and invasion of HCT116 cells, whereas knocking out ECHS1 inhibited the migration and invasion of SW480 cells.

Then, we detected lipid changes in CRC cells by changing the endogenous expression of ECHS1 only. As shown in Fig. 3D, after overexpression of ECHS1, the number of HexCer in HCT116 cells increased, whereas ceramide decreased. Consistent results were obtained in SW480 cells with ECHS1 knocked out. Further analysis focused on single-lipid metabolism. By taking the intersection of the increased phospholipid of the overexpression group and the decreased of the knockout group of ECHS1, we observed six kinds of lipids with positive correlation with ECHS1, including 2 BisMePA, 2 HexCer, 1 SM, and 1 Cer (Fig. 3F). Combined with the previous lipidomics results, we suspected that the dysregulation of ECHS1 regulated by LASP1 was related to ceramide glycosylation. UDP-glucose ceramide glycosyltransferase (UGCG) is a critical step to regulate the modulation of cellular activities by regulating the metabolism of ceramide and glycosphingolipids(GSLs) [20] (Fig. 3G). We then detected the expression of UGCG while endogenous ECHS1 changed. As shown in Fig. 3H, western blotting showed that the expression of UGCG and ECHS1 was synchronous, and ELISA indicated that the ceramide decreased when ECHS1 was knocked down (Fig. 3I), and it could be reversed when Eliglustat, a specific inhibitor of UGCG, was applied, which suggested that ECHS1 regulates UGCG to promote the ceramide to glycosylated ceramide.

### ECHS1 resists autophagy and apoptosis of CRC cells through the PI3K-Akt-mTOR pathway mediated by ceramide

**Fig. 4:**
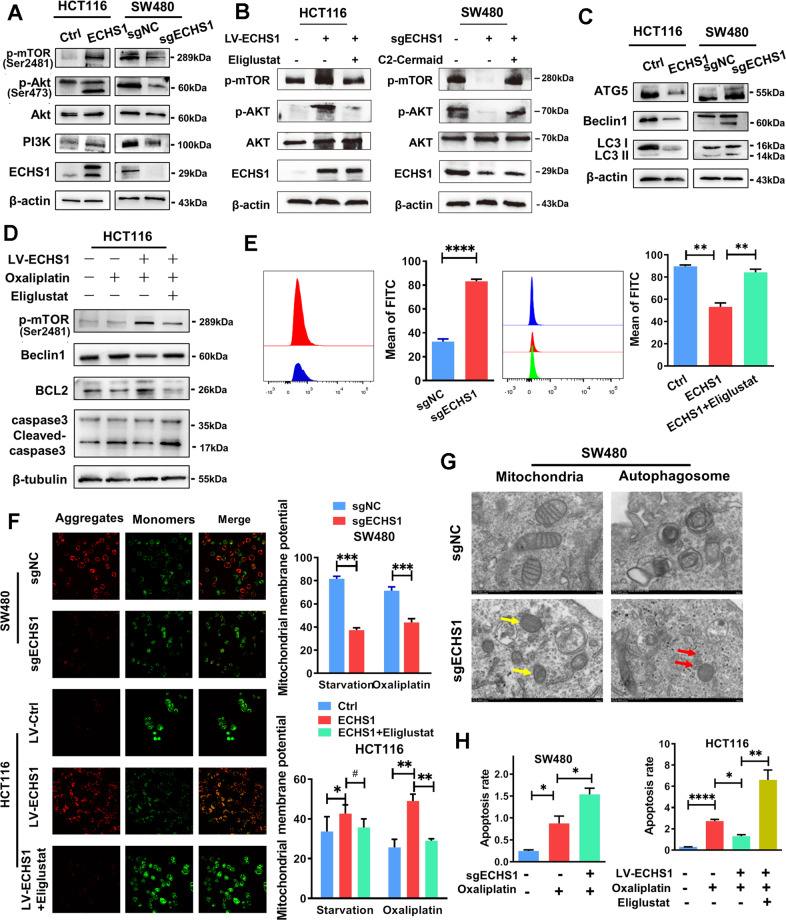
ECHS1 resists autophagy and apoptosis of CRC cells through the PI3K-Akt-mTOR pathway mediated by ceramide. **A** Western blot analysis of the expression of PI3K/Akt/mTOR pathway members (PI3K, Akt, phosphorylated AKT at Ser473, and phosphorylated mTOR) in ECHS1-overexpressing or ECHS1-knockout CRC cells. **B** Western blot analysis of the expression of PI3K/Akt/mTOR pathway members in ECHS1-overexpressing cells with the application of the inhibitor of UGCG eliglustat (left panel) or in ECHS1-knockout cells with the addition of C2 ceramide (right panel). **C** Western blot analysis of the expression of autophagy-related proteins (ATG5, Beclin1, and LC3II/LC3I). **D** Western blot analysis of phosphorylated mTOR, Beclin1, BCL2, and caspase3 in CRC cells with or without ECHS1 overexpression and Eliglustat (100 nmol). **E** Flow cytometry analysis of intracellular ROS content. Bars in the right panel represent the average fluorescence intensity. **F** IF assay analysis of JC-10 aggregates (red) and monomers (green) in the mitochondrial matrix with or without starvation (2 h) and Eliglustat (100 nmol). Bars of the right panel represent the ratio of the average fluorescence intensity of monomers (green) and aggregates (red). **G** Electron microscopy results show mitochondrial morphology (yellow arrows) and autophagosomes (red arrows) in ECHS1-knockout SW480 cells. **H** Histogram of flow cytometry assay showing the ratio of the average fluorescence intensity of apoptotic cells with or without Oxaliplatin (20 µg/µl) and Eliglustat (100 nmol).

One of the important mechanisms by which ceramide promotes apoptosis is to promote lethal autophagy by inhibiting the PI3K/Akt/mTOR pathway [21]. We doubt whether ECHS1 is related to this mechanism. As shown in Fig. 4A, Western blot assays showed that the PI3K/Akt/mTOR pathway in HCT116-LV-ECHS1 cells was reduced compared to that in the control group and activated when ECHS1 was knocked out in SW480 cells, and the opposite results were achieved. In the recovery experiment, whether the application of eliglustat, the inhibitor of UGCG, in ECHS1-overexpressing cells or the addition of C2 ceramide to ECHS1-knockout cells, the activation or inhibition of the PI3K/Akt/mTOR pathway could be reversed accordingly (Fig. 4B).

We also detected the expression of lethal autophagy-related proteins, such as ATG5, Beclin1, and LC3. As shown in Fig. 4C, the expression of ATG5 and Beclin1, autophagy-initiation-related proteins, was reduced, and the LC3II/LC3I ratio was increased. Furthermore, after the application of Eliglustat to inhibit the conversion of ceramide to glycosylated ceramide, the expression of the lethal autophagy-related proteins BCL-2 and Beclin1 and the apoptosis-related protein Caspase3 was restored (Fig. 4D). The release of reactive oxygen species (ROS) and the changes in mitochondrial membrane potential are usually thought to be important signatures of mitochondrial dysfunction-induced apoptosis [22]. Therefore, flow cytometry and immunofluorescence have been used to detect ROS and mitochondrial membrane potential. As shown in Fig. 4E and F, ROS production (Fig. 4E) increased and mitochondrial membrane potential (Fig. 4F) decreased when exogenous ECHS1 was overexpressed, which could also be reversed by Eliglustat. Furthermore, we observed the morphological changes of SW480sgECHS1 and the control group by electron microscopy. The results showed that after knocking out ECHS1, the mitochondrial double-membrane structure was lost, and the dorsal membrane was blurred. The number of autophagosomes near lysosomes also increased (Fig. 4G). Finally, we observed the effect of ECHS1 on drug-induced apoptosis of CRC cells. As shown in Fig. 4H, endogenous overexpression of ECHS1 reduced the drug-induced apoptosis of SW480 and HCT116 cells, and Eliglustat reversed this change.

### ECHS1 contributes to aggressive phenotypes and drug resistance of CRC cells both in vitro and in vivo

**Fig. 5:**
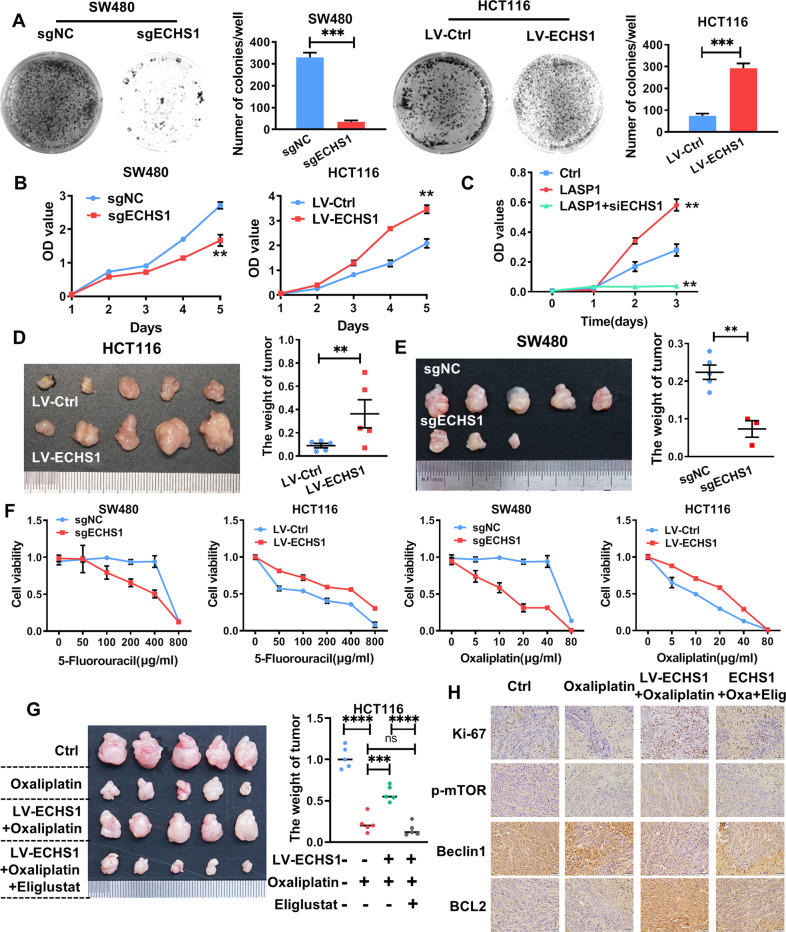
ECHS1 contributes to aggressive phenotypes and drug resistance of CRC cells, both in vitro and in vivo. **A** Representative figures of the clone-formation assay for the indicated cells. Bars on the right represent the number of formed clones. **B** CCK-8 assay for ECHS1 loss- and gain-of-function analysis of SW480 cells and HCT116 cells on CRC cell proliferation. **C** CCK-8 assay for LV-Ctrl-, LV-LASP1-, and LV-LASP1 plus siRNA-ECHS1-transfected RKO cells. **D**, **E** LV-Ctrl and LV-ECHS1 HCT116 cells (**D**) and sgNC and sgECHS1 SW480 cells (**E**) were injected subcutaneously into the backs of nude mice to access tumor growth. The representative figures of the tumors are shown. The right bar represents the weight of the tumors. The double asterisk (**) indicates *P* < 0.01. **F** CCK-8 assay analysis of cell viability with gradient application of 5-Fluorouracil and Oxaliplatin. **G** LV-Ctrl and LV-ECHS1 HCT116 cells were injected subcutaneously into the backs of nude mice with or without Oxaliplatin (1.5 mg/kg) and Eliglustat (60 mg/kg) to evaluate tumor growth. A representative figure of the tumors is shown. The right bar represents the weight of the tumors. **H** Subcellular localization of Ki-67, p-mTOR, Beclin1, and BCL2 in the indicated cells was assessed through immunohistochemical staining.

As shown in Fig. 5A and B, exogenous overexpression of ECHS1 dramatically enhanced the proliferation ability of HCT116 cells in CCK-8 and clone-formation assays. Meanwhile, knockout of ECHS1 inhibited SW480 cells. In addition, knocking down endogenous ECHS1 by siRNA restored the proliferation of CRC cells caused by LASP1 (Fig. S2, Fig. 5C). Additionally, in vivo subcutaneous tumor formation in BALB/C nude mice supported a stimulatory effect of ECHS1 on CRC cell proliferation (0.1 ± 0.05 g vs. 0.4 ± 0.3 g, P < 0.01; Fig. 5D), while knocking out ECHS1 dramatically inhibited the growth of subcutaneous tumors (0.2 ± 0.05 g vs. 0.08 ± 0.05 g, *P* < 0.01; Fig. 5E). We observed that endogenous overexpression of ECHS1 could reduce Oxaliplatin-induced apoptosis of CRC cells, and then we wanted to explore whether endogenous ECHS1 affected the drug sensitivity of cells. As shown in Fig. 5F, regardless of whether Oxaliplatin or 5-Fluorouracil was applied, the survival rate was significantly increased in ECHS1-overexpressing cells and decreased in ECHS1-knockout cells compared with the corresponding control cells. Furthermore, in vivo subcutaneous tumor formation in BALB/C nude mice was significantly increased in the ECHS1-overexpressing groups compared with the control groups after the injection of Oxaliplatin (1.5 mg/kg). Administration of Eliglustat (60 mg/kg) reversed ECHS1-induced drug resistance and subcutaneous tumor growth (Fig. 5G). Meanwhile, as shown in Fig. 5H, immunohistochemical staining demonstrated that the expression of p-mTOR and the mitochondrial membrane stability marker BCL2 was increased when the autophagy-related indicator Beclin1 was decreased in the HCT116-LV-ECHS1 group compared with the HCT116-Ctrl group. Meanwhile, along with the increase in ECHS1 expression, the number of Ki-67-positive tumor cells significantly increased in tumors. More importantly, application of Eliglustat (60 mg/kg) reversed the expression of pathway proteins activated by ECHS1.

### Overexpression of ECHS1 is associated with tumor progression and poor prognosis of CRC

**Fig. 6:**
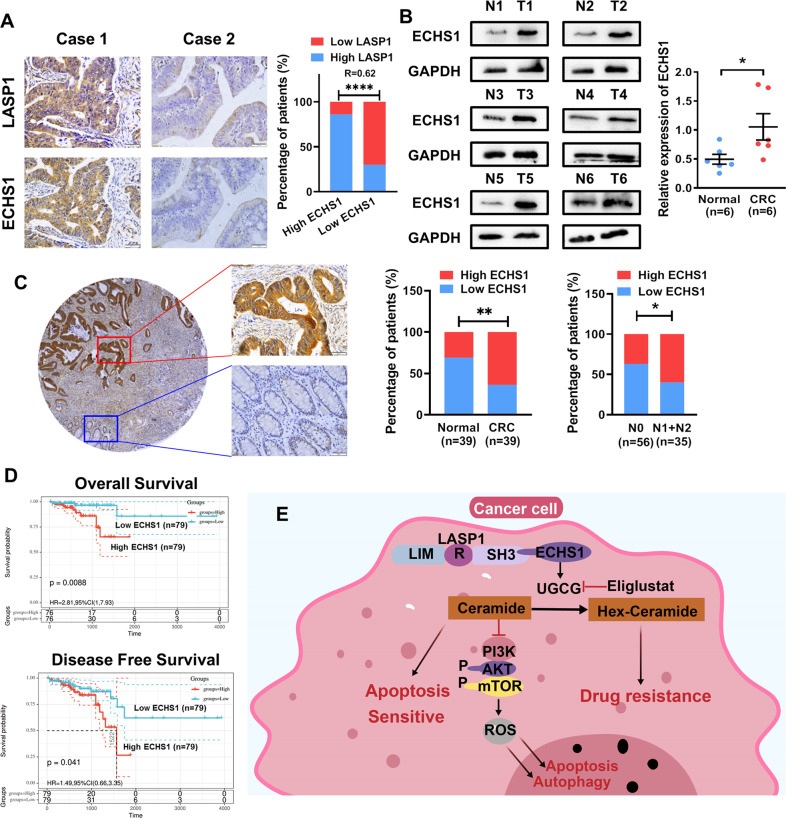
Overexpression of ECHS1 is associated with tumor progression and poor prognosis of CRC. **A** Representative images of IHC staining analyses of LASP1 and ECHS1 in CRC tissues. **B** Western blot analysis of ECHS1 in CRC tissues (T) and adjacent nontumor tissues (N). The scatterplot on the right shows the relative expression of ECHS1 in normal and CRC tissues (normalized to GAPDH, *P* < 0.05). **C** Left panel: immunohistochemistry (IHC) analysis of ECHS1 in CRC tissues and adjacent nontumor tissues. Right panel: histogram representing IHC results in CRC tissues (T) and adjacent nontumor tissues (N) and with or without lymph-node metastasis. **D** Kaplan–Meier survival curves and univariate analyses (log rank) for CRC patients with distinct expression levels of ECHS1. **E** Sketch map of the mechanism of ECHS1-mediated CRC proliferation and drug resistance.

As shown in Fig.6A, the immunohistochemical results showed that relatively high expression of ECHS1 was often observed in CRC samples overexpressing LASP1 (*R* = 0.62, *P* < 0.0001). Next, by detecting the expression of ECHS1 in six CRC tissues, the western blot assay results showed that the expression of ECHS1 was overexpressed in CRC tissues compared with matched nontumor tissues (Fig. 6B). Immunohistochemistry further demonstrated that the overexpression of ECHS1 was significantly upregulated in 64% (25/39) of the CRC tissues compared with 30% (12/39) of the adjacent normal tissues (Fig. 6C, *P* < 0.001). IHC also demonstrated that ECHS1 overexpressed at a much higher rate in CRC tissues with distant lymph-node metastasis (N1 + N2) (Fig. 6C, *P* < 0.05). Kaplan–Meier survival analysis of a previously published CRC dataset (TCGA-READ, *n* = 79) revealed that ECHS1 expression is closely correlated with patient overall survival (OR) and disease-free survival (DSS). Patients with low ECHS1 expression had a significantly better prognosis (Fig. 6D).

## Discussion

LIM and SH3 protein 1 (LASP1) has been found overexpressed in the amount of tumors including breast cancer [3] and ovarian cancer [4]. According to our previous study, LASP1 was highly overexpressed in CRC tissues, which is closely associated with lymph nodes and distant metastases and the poor prognosis of patients [6]. In this study, we first found that LASP1 is related to the metabolism of phospholipids, especially ceramides, in tumor cells, and regulates the lipid-metabolism enzyme ECHS1. Simultaneously, ECHS1 tends to interact with the SH3 domain at the C terminus of LASP1 to stimulate ceramide metabolism and cancer aggressiveness. As for the mechanism behind LASP1-driven prevention of ECHS1 degradation in the proteasome, interaction of LASP1 and HSPA1A had been reported in neck squamous cell carcinoma [23]. HSPA1A, which is also known as HSP70, was widely found in the surface of the lysosome membrane, and protected its chaperone protein from the ubiquitin–lysosomal-degradation pathway by inhibiting lysosomal permeability [24]. Moreover, many studies had shown that HSP70 was not only involved in the ubiquitination and subsequent degradation by dissociating 26 S proteasome complexes (into free 20 S proteasomes and bound 19 S regulators), preserving 19 S regulators, and reconstituting 26 S proteasomes [25], but also could be cleaved independent of ubiquitination by the 20 S proteasome since HSP70 itself was a substrate of 20 S proteasomes [26]. Therefore, LASP1 may regulate the proteasome-degradation pathway via its chaperone protein.

ECHS1 is a key enzyme that catalyzes the second step of the β-oxidation pathway in fatty-acid metabolism. Apart from its critical roles in regulating fatty acid metabolism, numerous literatures have indicated that ECHS1 might be involved in the development of tumor, including colon [14, 15], liver [27], gastric [17], and renal [28] cancer. In CRC cell lines, it has been reported that ECHS1 is able to suppress proliferation and migration through PI3K–Akt–GSKβ signaling pathways [15], and the knockdown of ECHS1 attenuates HCC proliferation by impairing cell metabolism and inducing cell apoptosis and autophagy by activating the AMP protein kinase (AMPK) pathway [16]. Additionally, in the human gastric cancer cell lines, the protein levels of ECHS1 were significantly higher than those in nonneoplastic gastric epithelial mucosa cells [17]. Constitutive knockdown of ECHS1 significantly inhibited cell proliferation and migration through the PI3K–PKB and GSK3β signaling pathways. Although several studies have shown that ECHS1 expression is absent in clear-cell renal-cell carcinoma (ccRCC) [28] or CRC [29], Western blot and IHC assays clearly showed that the expression of ECHS1 is highly expressed in colon-cancer cells and tissues and associated with advanced progression and poor prognosis based on our study. Overexpression of ECHS1 could promote tumor-cell invasion, migration, and proliferation of CRC cells in vitro and in vivo. Upon further detection of cell-lipid metabolism by LC–MS, except for the well-known function of ECHS1 in regulating FAO, we found that after overexpression of ECHS1, the content of ceramide decreased, while glycosylated ceramide increased. The results of the Western blot assay showed that ECHS1 could regulate UGCG, which catalyzes the first step of ceramide glycosylation to convert ceramide to glucosylceramide in sphingolipid metabolism. Thus, we suspect that ECHS1 affects the phospholipid metabolism–glycosylation of ceramide in CRC cells by regulating UGCG in CRC cells. Although numerous studies have demonstrated abnormal metabolism of phospholipids in CRC progression, the underlying mechanism remains unclear.

Ceramide (Cer) is a tumor suppressor that can enhance the signal-initiating apoptosis, autophagy, and cell-cycle arrest, and its reduction helps prolong the survival time of cancer cells [30]. Mechanistic research clarified that ceramide triggers autophagy by regulating the mTOR signaling pathway and interfering with Beclin 1:BCL-2 complex in a phosphorylation-dependent manner mediated by c-Jun N-terminal kinase 1 (JNK1) [31]. Western blot results showed that the PI3K/Akt/mTOR pathway was suppressed and that autophagy-related proteins, such as ATG5, Beclin1, and LC3II/LC3I, were activated when ECHS1 was knocked out in CRC cells, which could be resorted by Eligustat, an inhibitor of UGCG. Thus, flow cytometry and immunofluorescence assays showed that ROS production increased and mitochondrial membrane potential decreased when exogenous ECHS1 was knocked out, in which an important mechanism of the mitochondrial pathway is leading to cell apoptosis. In summary, ECHS1 resists apoptosis and autophagy of CRC cells through the ceramide-mediated PI3K–Akt–mTOR pathway.

Evidence suggests that the sphingolipidome plays an important role in cancer-drug resistance. It has been reported that in the human ovarian carcinoma-cell lines, application of drugs such as Taxol could change the phospholipid metabolism of tumor cells to activate SMase to generate Cer [32]. Similarly, under the induction of drugs, lung-cancer cell A549 produces more dihydroceramide and ceramide through de novo synthesis than matched drug-resistant cell lines [33]. Different from Cer and SM, glycosphingolipids, including LacCers and HexCers (GalCers and GluCers), are generally regarded as an important biomarker for maintaining tumor growth and promoting chemotherapy resistance [34]. Glucosylceramide synthase has been observed upregulated in a series of tumors after drug treatment, which suggests that glycolipids may be involved in promoting tumor-drug resistance [35]. In CRC cells, GalCer and LacCer had been reported that characteristically increased in Oxaliplatin-resistant cells and the overexpression of UGCG gene is significantly associated with the decrease in disease-free survival of patients with colorectal cancer, which suggests that glycosylation plays a pivotal role in Oxaliplatin chemosensitivity [36].

In this study, we found that both in vivo and in vitro ECHS1 promoted UGCG-mediated ceramide glycosylation, which led to drug resistance, including 5-Fluorouracil and Oxaliplatin, in CRC cells. More importantly, the application of Eliglustat, an oral UGCG inhibitor, could reverse the drug resistance caused by ECHS1, which provides a new target for chemotherapy of CRC.

## Conclusions

In summary, we found that LASP1 is associated with sphingomyelin metabolism in CRC cells and identified ECHS1 as a new LASP1-interacting protein. ECHS1 regulates UGCG-mediated ceramide glycosylation, which further stimulates CRC progression and resists apoptosis and autophagy through the PI3K/Akt/mTOR pathway. Furthermore, the application of Eliglustat could restore chemotherapeutic drug resistance caused by ECHS1-mediated phospholipid metabolism disorders (Fig. 6E). The current research illustrates ECHS1 as a novel predictive biomarker and provides a new insight for clinical CRC chemotherapy resistance.

## Supplementary information

Supplementary figures

## Data Availability

The datasets generated and/or analyzed during the current study are not publicly available but are available from the corresponding author on reasonable request.
