# Supplementary material for: ECHS1, an interacting protein of LASP1, induces sphingolipid-metabolism imbalance to promote colorectal cancer progression by regulating ceramide glycosylation
Source: Cell Death Dis. 2021 Oct 6;12(10):911. doi: 10.1038/s41419-021-04213-6 (PMC8494735; doi:10.1038/s41419-021-04213-6)
Supplement: Supplementary file 1 — Supplementary figures [file 41419_2021_4213_MOESM1_ESM.docx]

**Supplementary figures**

**
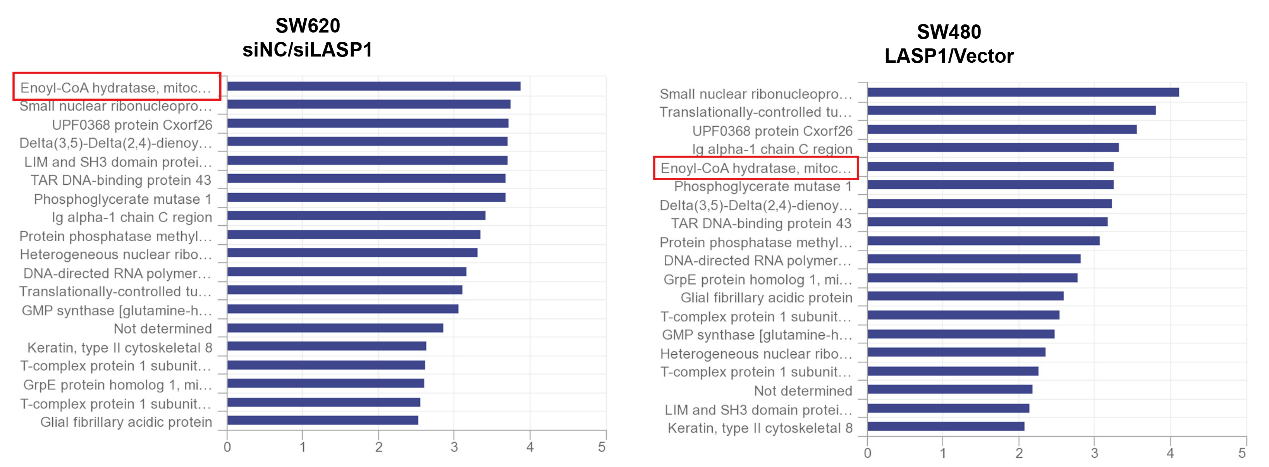
**

**Figure S1. The histogram showed fold change of 19 differentially expressed proteins in LASP1 knocking-down SW620 cells (left panel) and LASP1** **overexpressed SW480 cells (right panel) (foldchange >2, p<0.01) .**

**
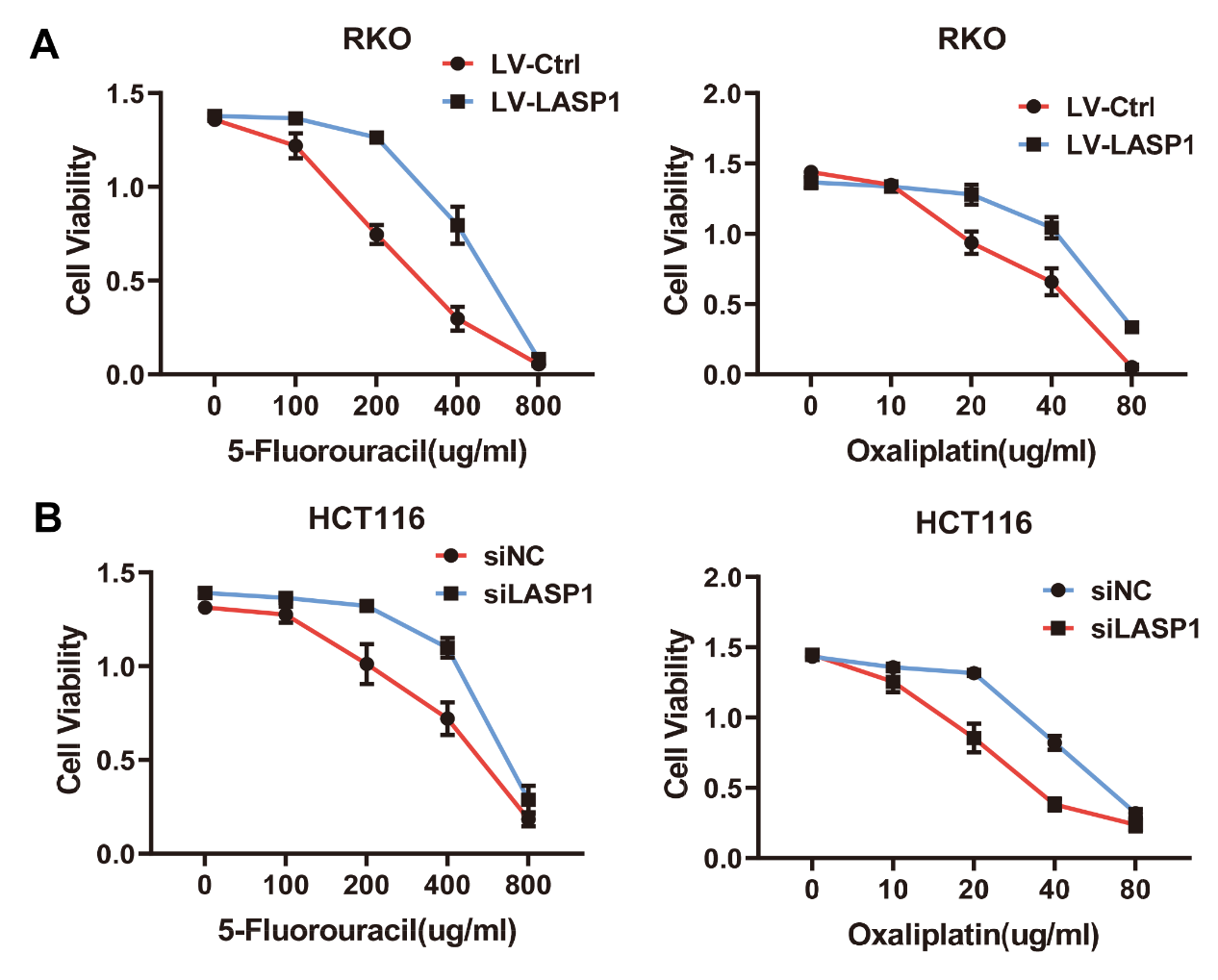
**

**Figure S2. CCK-8 assay analysis of cell viability in indicated RKO cells (A) and HCT116 cells (B) with gradient application of 5-Fluorouracil and Oxaliplatin.**
